# The role of nature in cancer patients' lives: a systematic review and qualitative meta-synthesis

**DOI:** 10.1186/s12885-017-3366-6

**Published:** 2017-05-25

**Authors:** Sarah Blaschke

**Affiliations:** 1 Cancer Experiences Research, Peter MacCallum Cancer Centre, 305 Grattan Street, Parkville, Melbourne, 3000 Australia; 2 Sir Peter MacCallum Department of Oncology, Faculty of Medicine, Dentistry and Health Sciences, The University of Melbourne, Parkville, Australia

**Keywords:** Cancer, Nature, Supportive care, Patient resources, Attachment theory, Meta-synthesis

## Abstract

**Background:**

A systematic review and meta-synthesis was conducted to identify, compare and synthesize the published qualitative literature contributing to our understanding of the role of nature in cancer patients’ lives.

**Method:**

An electronic search of Medline, CINAHL, PsycINFO and Cochrane Databases was conducted to identify qualitative studies focused on cancer patients’ nature experiences published between January 1985 and May 2015. Records were assessed according to pre-defined inclusion criteria. Data were extracted on study characteristics and evaluated using the COREQ guidelines for comprehensive quality reporting. Qualitative data from ‘results’ and ‘findings’ sections were entered into data management software NVivo in order to identify recurring themes and facilitate interpretation across studies.

**Results:**

From 11 eligible publications, seven inter-related core themes with descriptive themes were identified as follows: connecting with what is valued; being elsewhere, seeing and feeling differently; exploration, inner and outer excursions; home and safe; symbolism, understanding and communicating differently; benefitting from old and new physical activities; and, enriching aesthetic experiences.

**Conclusions:**

Nature provides patients with unburdened physical and psychic space invested with personal significance. Findings propose nature’s role as a “secure base” offering patients a familiar and nurturing context from which new perspectives can emerge and caring connections can be made with themselves, others, the past, and the future. As such, nature supported patients to navigate the clinical and personal consequences of cancer. Comprehensive representation of cancer patients’ nature experiences identified patient values and care opportunities embedded in clinical and personal environments, which may be considered for future research and care service development.

**Electronic supplementary material:**

The online version of this article (doi:10.1186/s12885-017-3366-6) contains supplementary material, which is available to authorized users.

## Background

The global increase of cancer incidence will soon impact at least one in three people, either personally or through a relative or friend [1, 2]. Reducing the burden of cancer and supporting those affected by cancer has become a healthcare priority demanding cost-effective and high quality solutions. These solutions need not only treat cancer, but also provide personalized care, prevent disease and maintain or even improve patients’ quality of life for as long as possible. In light of these shifting healthcare demands, research is helping to illuminate aspects of cancer patients’ experiences in order to develop improved care services.

Renewed interest in nature’s impact on human health and well-being is evident in burgeoning research on the subject across various disciplines such as public health [3], environment and behaviour [4], planning and design, and environmental disciplines [5]. Although the subject is diversely conceived, emerging research shows positive associations between nature–human interactions and health promotion [6], as well as disease prevention [7]. Preliminary investigations into nature-based interventions across several healthcare contexts suggest their potential to ease illness related strain [8] and to impact positively on patients [9]. Keniger has categorized the broad spectrum of nature experiences into three levels of engagement offering a productive frame for delineating different types of nature-based interventions [10]. They are as follows: 1) indirect engagement, e.g. views to nature and art depicting nature [11]; 2) incidental engagement, e.g. walking and resting outdoors [12]; and 3) intentional engagement, e.g. outdoor adventure therapy [13]. These nature-based interventions are being trialled with mixed clinical populations across three types of settings including: indoor settings, e.g. intensive care units [14]; organized and designed outdoor environments, e.g. rehabilitation gardens [9]; and remote or wilderness sites, e.g. forest settings [15].

As a relatively new field of empirical inquiry, health-nature research has favoured an evidence-based interventionist paradigm with the aim to parallel the judicious procedures of medical research and resemble the system it intends to adopt. Consequently, inquiry has prioritized research instruments, questionnaires, and audits designed a priori by researchers assuming that quality of experience can be measured quantitatively. There is little in the literature to illustrate the role of human-nature interactions in patients’ lived experiences of health and disease and whether or not, from their perspective, nature contributes to recovery, health and well-being. In the cancer setting, various bio-psychosocial challenges have been identified that impact patients’ daily living needs [16, 17]. Research has found unmet supportive care needs related to physical functioning, information, and emotional care for both urban and rural patients [18]. Given the commonplace availability of nature in urban and rural environments, as well as in some clinical settings, numerous opportunities exist for cancer patients to potentially utilize and benefit from contact with nature. To date, only one narrative review of the literature on cancer survivors’ nature-based experiences could be located [19], which demonstrates a need for deeper and broader understanding of nature’s relevance across the cancer journey. To the author’s best knowledge, there is no published meta-synthesis of findings concerning the role of nature in cancer patients’ lives. Two important questions remain open for investigation, which according to Sandelowski are at the heart of practitioners’ and patients’ concerns: “a) Does it work? and b) If it works, should it be used?” [20] (p.1368).

This meta-synthesis underpins a larger investigation, which aims to determine feasible and appropriate solutions to incorporating nature-based care opportunities in cancer care contexts. Its specific aim, and its contribution to this larger body of work, is to explore and identify the various roles of nature in the lives of cancer patients and to explore how these experiences support or detract from their recovery processes (i.e. *does it work?*), and if patients value and seek these opportunities (i.e. *should it be used?*). Synthesis of published qualitative research has been described as integrating research evidence to reach new theoretical understandings of a chosen topic [21]. Accordingly, this review aimed to contribute a new conceptual representation of the existent body of knowledge grounded in an interpretative framework [22]. Synthesized understandings can make findings more accessible to key stakeholders such as healthcare leaders and practitioners, policymakers, researchers, and consumers who require translatable knowledge if nature is to be safely and effectively incorporated into supportive care. To assist this process, the following research question was addressed: What does the published qualitative research literature contribute to understanding the role of nature in cancer patients’ lives?

## Methods

Meta-synthesis is the examination, critical comparison and synthesis of published qualitative studies that concern a common topic [21–23]. It is a validated research process [24] that aims to gain a fuller knowing of a phenomenon than would be achieved from a single, isolated study [22]. Based on previously published meta-syntheses [23, 25], the present review followed a multi-stage approach comprising: 1) determining the review focus; 2) identification of published and relevant literature; 3) quality appraisal of the included documents; 4) data extraction and identification of key concepts grounded in the raw data contributed by research participants; and 5) development and comparison of core themes across the documents and their synthesis into a new conceptual representation. Each of these stages is detailed below.

### Determining the review focus

The review focus was determined by the author’s doctoral thesis topic, which concerns the investigation of nature’s role in cancer patients’ lives and aims to determine nature-based care opportunities in cancer care contexts. The present paper reports research carried out by a sole researcher. The strategies employed to mitigate reporting bias are presented in the Limitations section below. For the purposes of this review, nature was defined as the phenomena of the physical world collectively, including various forms of vegetation and habitat, natural and humanly designed landscapes, natural cycles, processes, weather, wildlife and domestic animals, and other features and products of the earth including man-made creations which creatively organize and depict these nature elements [6, 26–28].

The Cochrane Database for Systematic Reviews and the International prospective register of systematic reviews PROSPERO were first searched to ensure no identical or similar review was underway. The review was initially registered with PROSPERO as a systematic review of nature-based intervention research, however the literature searches returned insufficient studies addressing primary outcomes, which is a requirement for PROPERO registration. Consequently, the review focus shifted in order to address the available qualitative research, rather than intervention research. Registration (CRD42014015291) was withdrawn in February 2016.

### Identification of published and relevant literature

A comprehensive systematic search of the published literature was conducted in following electronic database: Medline, CINAHL, PsycINFO and Cochrane Database of Systematic Reviews from January 1985 to March 2015. A research librarian was consulted for developing search strategies for the respective databases, which included combinatorial strings of Subject Headings and text word searches containing terms related to: “cancer” and “nature” (see Additional file 1 Search protocol). Electronic searches were supplemented by manual search of two relevant journals: AHTA Journal of Therapeutic Horticulture and ACTAHORT. Non-peer-reviewed articles retrieved from the electronic searches were read for the purpose of searching reference lists. The inclusion criteria for publications were: full-text, peer-reviewed journal articles published in English language, which included primary qualitative data of empirical studies conducted with cancer populations.

Initial searches retrieved titles and abstracts only. Duplicates, obviously irrelevant studies, and studies that did not meet the above inclusion criteria were removed. The next stage of the selection process was directed by reading records at full-text level and identifying whether the studies reported the use of qualitative methods to explore the experiences of contact with nature from the perspective of individuals who had experienced cancer. The study’s specific qualitative method employed or the researchers’ philosophical positioning (e.g. phenomenology, grounded theory, ethnography) were not directive because their shared focus is understood to be the elucidation of meaning and processes of a given phenomenon from the perspective of the experiencer through interpretive means [29]. Therefore, studies based on a predominantly quantitative research design with a minor qualitative supplement were not included as their aim precluded relevant data and interpretation to explore participants’ personal experiences.

Studies were eligible if the sample included people who had directly experienced cancer; excluded were studies reporting only from the perspective of caregivers or healthcare professionals. Studies focusing on related topics such as post-occupancy evaluations of hospital gardens [30] and physical exercise research [31], not exploring nature experiences were excluded.

### Quality appraisal of the included documents

Research synthesists are responsible for appraising the quality of included studies in order to report transparently on their validity and generalizability. Studies with methodological weakness will negatively impact on the strength of the conclusions in a meta-synthesis. Methodological discussion exists questioning the adoption of prescriptive evaluation protocols based on quantitative ontology and epistemology for evaluating qualitative research [32, 33]. In response, instruments are being developed to better reflect appraisal criteria relevant to qualitative research such as confirmability, dependability, and credibility [34]. Examples are the Joanna Briggs Institute Qualitative Assessment and Review Instrument (JBI-QARI) [35] and the Critical Appraisal Skills Programme (CASP) [36]. However, the use of prescriptive criteria remains controversial [33]. Sandelowski and colleagues recommend not excluding papers based on quality checklists, but to focus instead on topical relevance and to employ broad evaluation criteria for the final quality reporting of included studies [29]. Taking this discussion into account, the present meta-synthesis prioritized the following initial question to gauge topical relevance: Does this publication contribute genuine qualitative data exploring cancer patients’ nature experiences? The Consolidated Criteria for Reporting Qualitative Research (COREQ) guidelines [37] were considered an appropriately broad framework for assessing the quality of included studies, and they were also used as a guide for reporting findings in this present meta-synthesis. The strength of the COREQ guidelines lies in outlining three general domains of methodological rigour applicable to various qualitative methods, as demonstrated in one successful example of meta-synthesis by Luker and colleagues [38].

1. Research reflexivity: information on the researchers’ background, biases, and relationship with the participants (confirmability).
2. Study design: appropriate methods for data collection and documentation, information regarding sampling and recruitment, description of study setting and context (dependability).
3. Analysis and reporting: evidence of raw data, clarity of interpretive process, consistency of raw data and findings (credibility).

### Data extraction and identifying key concepts

Author SB recorded data on the following characteristics of included studies: reference details (year of publication, author, first author’s country of origin); discipline; sample size; participant characteristics; methodological approach; data collection method; and focus of the study. All data presented as ‘results’ or ‘findings’ were entered into data management software QSR International’s NVivo 10 for Mac [39]. This included primary data (e.g. participant quotes) as well as authors’ interpretations (e.g. thematic description). All unaltered textual material was read in order to gain a general understanding of the material before inductively coding data line-by-line with the aim to glean salient underlying concepts. In this process, for example, the statement ‘escape from the fear and worry associated with cancer, a place that was safe’ [40] was reduced and captured with the descriptive label (code) ‘safe refuge’. Next, codes were grouped into meaningful clusters from which prominent descriptive themes emerged, for example, the codes ‘losing the capacity for gardening is painful’ [41], and ‘losing bond with garden’ [40] were grouped as ‘losing connection’. This interpretive task reflects terminology and descriptions based on the researcher’s own understanding of the material and is presented in Additional file 2 Themes and illustrative quotes, which includes reference to raw data in order to enable the reader’s own appraisal of interpretative and conceptual congruity.

### Thematic development and synthesis

Using a constant comparison approach [42], common events and attributes of the studied phenomenon were identified and careful attention was paid to the frequency of recurrence across different studies, which strengthens emerging themes [24]. For example, the descriptive theme ‘connecting with something outside’ arose in nine studies [13, 27, 28, 40, 41, 43–46], while ‘stimulating sensory experiences’ was found in only four [27, 28, 44, 46]. Once the entire dataset was scrutinized (primary data and authors’ interpretations) and no more themes could be teased out, the researcher returned to the included publications for a second narrative reading in order to confirm contextual relationships between the themes. This informed the final theoretical synthesis of findings into overarching, analytical themes, or ‘core themes’ [47]. These core themes were developed in relation to the meta-synthesis’ main objective, which gave rise to a hierarchical pattern of core and descriptive themes organized according to their theoretical depth and relevance.

## Results

### Identification of published and relevant literature

**Fig. 1:**
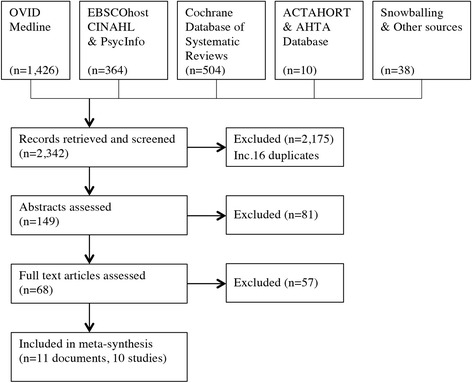
Flowchart of search and selection process

The systematic search identified 2342 records, 149 of which remained after duplicates and obviously irrelevant records were removed (Fig. 1). A further 77 of these records were eliminated by asking the question: Does this contribute genuine qualitative data exploring cancer patients’ nature experiences? The 68 remaining articles were read in full to determine the sample characteristics, the type of qualitative method, and whether the focus was indeed to explore nature experiences. Seventeen articles were identified as having substantial relevance and were further appraised against COREQ quality criteria. One of these articles was omitted because its study design was primarily quantitative [48]. Four studies lacked sufficient primary data and methodological rigour to produce understandings grounded in participants’ perceptions [49–52]. Finally, one study did not differentiate clearly between the views of cancer patients and health professionals [53]. Overview of reasons for exclusion is provided in Additional file 3 Excluded publications. The 11 remaining documents were accepted for synthesis comprising ten articles (nine separate studies) and one thesis.

### Characteristics of included documents

Table 1 summarizes the characteristics of the included documents. Studies were published between 2000 and 2014 with the majority (8/11) published after 2005. Data were contributed by 240 cancer patients across the lifespan with varying diagnoses and included survivors and palliative patients. Sample size ranged from 3 to 88, with seven studies reporting a sample size <16. Studies were conducted in four countries: United States (US) (*n* = 4), Canada (*n* = 4), United Kingdom (UK) (*n* = 2), and Australia (*n* = 1). Two publications by Unruh [40, 41] reported on different aspects of one data set collected in a single study.

**Table 1:** Characteristics of included studies

First author (year) country (ref)	Discipline	Sample size, n	Participants [ages]	Methodological approach	Data collection	Focus
Blair (2013) US [43]	Nutrition Science / Oncology	12	Survivors mixed diagnosis [adult mean = 56 yrs., children mean = 10 yrs]	Not stated	Semi-structured telephone survey	To explore the feasibility and effects of a vegetable gardening intervention pairing 12 adult and child cancer survivors with Master Gardeners.
Butterfield (2014) UK [28]	Doctoral thesis in Arts	88	Mixed diagnosis [not stated]	Ethnography	Interviews (20 min) post photo-elicitation, open-ended online survey	To explore experiences of cancer care centres’ gardens and how these support quality of care and a sense of wellbeing.
English (2008) Canada [54]	Public health	14	Breast cancer patients at various stages [30–65 yrs]	Grounded Theory	Semi-structured in-depth interviews	To explore the ways in which environments contribute to healing and recovery from breast cancer.
Pascal (2010) Australia [55]	Public health	15	Breast cancer patients at various stages [35–70 yrs]	Hermeneutic phenomenology	In-depth interviews	To explore how therapeutic landscapes shape the experiences of living with cancer.
Ray (2013) Canada [44]	Community health	15	Breast cancer survivors [14 of 15 participants were 50 yrs. or older]	Content analysis	Semi-structured interviews	To explore breast cancer survivors’ lived experience of dragon-boating and how it influences health-related quality of life.
Rowlands (2008) UK [11]	Palliative care	12	Advanced cancer inpatients [25–65 yrs]	Phenomenology	Semi-structured interviews	To explore the views of advanced cancer patients about the effect of the ward environment on their overall well-being.
Spees (2015) Canada [45]	Health and rehab	28	Survivors [35–83 yrs]	Thematic analysis	Focus group	To explore perceptions of health behaviour change in cancer survivors following an urban gardening program.
Stevens (2004) Canada [13]	Nursing and medicine	11	Adolescent cancer patients [ages not given]	Naturalistic	Videotaped interviews during expedition	To explore adolescent cancer patients’ experiences in an adventure therapy program from a health-related quality of life (HRQOL) perspective.
Unruh (2000) US [46]	Occupational therapy	3	Breast cancer patients [35–57 yrs]	Thematic analysis	Semi-structured interviews	To explore the meaning of gardening for three breast cancer patients using attention restoration theory.
Unruh (2002) US [40]	Occupational therapy	18 / 42	Mixed cancer diagnosis / other chronic disease [32–80 yrs]	Phenomenology	Semi-structured interviews	To explore the meaning of gardens and gardening in the lives of people with serious health problems.
Unruh (2011) US [41]	Health and human performance	15 / 42	Mixed cancer diagnosis / other chronic disease [32–80 yrs]	Hermeneutic phenomenology	Semi-structured interviews	To explore the meaning of gardens and gardening as a lived experience of leisure gardening amongst people with serious health problems, chronic and progressive disease.

### Quality appraisal

The study designs and methodological rigour of the included articles were variable in quality. Notably, all articles omitted information about the researchers’ reflexive practice. As mentioned above, it has been argued that checklists may be an inappropriate instrument for evaluating qualitative research; however, the COREQ guidelines were used in this review as a broad guide for identifying unacceptably deficient methodology and provided a structured approach for consistent and fair treatment of dissimilar studies. The governing authority, nonetheless, remained the study’s subject relevance and whether it contributed genuine qualitative data. For example, according to the COREQ 32-item checklist, Rowlands’ [11] study scored only 11/32, yet it was found to contribute valuable insight about hospitalized cancer patients’ perceptions of connecting with the outside world when viewing nature through a window.

Based on the COREQ appraisal framework, a general comment can be made about the included studies regarding their consistent methodological weakness in the first domain (researcher’s reflexivity). In total, all studies combined scored 25/88 in this domain. Only two studies [41, 44] explained audit trails and strategies for verifying data analysis (participant checking). Overall, studies were rated considerably low in all three domains of the COREQ, and only three [13, 28, 41] scored individually >21 out of 32 appraisal items. Scoring appears in Additional file 4 Quality assessment.

### Synthesis

The studies described a spectrum of nature experiences including views to nature from within the hospital [11], contact with therapeutically designed and natural landscapes [28, 54], domestic gardening and structured gardening programs [40, 41, 43, 45, 46], retreats in natural environments [55], dragon-boating [44], and outdoor adventure programs [13]. The initial coding process provided a broad sense of the material and explicated salient concepts. It was notable that individual researchers deployed different interpretative language when dealing with similar ideas and concepts, for example: experiences alluding to relaxation in nature were reported by English and colleagues as, ‘nature appears to inspire feelings of calmness’ [54]; while Unruh and colleagues reported, ‘Worried minds were eased by thinking about the garden’ [46]; and yet another study expressed the concept of relaxation as, ‘providing reprieve from the everyday stresses’ [45]. Consequently, this meta-synthesis prioritised primary data (participants’ own words) where available in order to start from, and remain close to, original formulations during the coding process. However, all extracted findings including individual authors’ interpretations were considered. Grouping of codes into logical clusters generated 22 descriptive themes. The shared and interrelated meanings of these descriptive themes informed the development of seven core themes: connecting with what is valued; being elsewhere; exploration, inner and outer excursions; safe and home; symbolism, understanding and communicating differently; benefitting from old and new physical activities; and, enriching aesthetic experiences. The core themes *connecting with what is valued* and *being elsewhere, seeing and feeling differently* pervaded all studies and the remaining five core themes were identified consistently in at least seven documents. Table 2 presents the thematic findings with citation to source documents and is followed by text summarizing each core theme with reference to descriptive themes in italics.

**Table 2:** Themes and source documents

Core themes	Descriptive themes	Source documents
1	Connecting with what is valued	[11, 13, 27, 28, 40, 41, 43–46, 55]
	* Connecting with something outside*	[13, 27, 28, 40, 41, 43–46]
	* Connecting with Self*	[2, 7, 10, 13, 15, 28, 44]
	* Connecting with others*	[13, 28, 40, 41, 44–46]
	* Connecting with nature*	[11, 13, 27, 28, 41, 44, 46, 55]
	* Connecting with the past, reminiscing and remembering*	[13, 28, 40, 41, 45, 46]
	* Losing connection*	[40, 41, 46]
2	Being elsewhere, seeing and feeling differently	[11, 13, 27, 28, 40, 41, 43–46, 55]
	* Gaining distance (break) from everyday strain*	[27, 28, 40, 41, 44–46, 55]
	* Contrasting the clinical experience*	[13, 27, 28, 46, 55]
	* Visual escape, a different way of being elsewhere*	[11, 27, 28, 40, 46]
3	Exploration, inner and outer excursions	[13, 27, 28, 40, 41, 43–46, 55]
	* Exploring the distant and extraordinary*	[13, 27, 41, 44, 46, 55]
	* Exploring future scenarios*	[13, 28, 41, 46]
	* Exploring new ideas, behaviors and activities*	[13, 44–46]
	* Exploring the caregiver’s role, caring for the garden*	[40, 41, 43, 45, 46]
4	Home and safe	[13, 27, 28, 40, 41, 45, 46, 55]
	* Domestic scale*	[28, 40, 41, 45, 55]
	* Caring and being cared for*	[27, 28, 40, 41]
	* Supportive infrastructure*	[13, 27, 28, 45, 46]
5	Symbolism, understanding and communicating differently	[13, 27, 28, 40, 41, 46, 55]
	* Using metaphors found in nature*	[13, 27, 28, 41, 46, 55]
	* Reflecting and mirroring inner and outer life*	[13, 27, 28, 40, 41, 46, 55]
6	Benefitting from old and new physical activities	[13, 27, 40, 43–46]
	* Enjoying new and old activities in nature*	[27, 40, 43–46]
	* Benefitting from being active in nature*	[13, 28, 40, 43–46]
7	Enriching aesthetic experiences	[13, 27, 28, 43, 44, 46, 55]
	* Stimulating sensory experiences*	[27, 28, 44, 46]
	* Aesthetic enrichment*	[13, 27, 28, 43, 44, 55]

#### Connecting with what is valued

Every study reported on connections facilitated by nature, which participants valued and, in some instances, had to let go of due to their cancer experience. Participants consistently sought connection with something ‘emotionally uplifting’ [54] and *outside their daily experiences* of diagnosis and treatment. The ‘importance of contact with the outside world, especially nature’ [11] was noted as supportive in different ways, for example: ‘to transform emotional and psychological health’ [54] and for creating ‘a more optimistic outlook on life in general’ [43].

Patients found nature useful for establishing and maintaining *connection with themselves* and for reflection ‘on their own state of being’ [28]. One breast cancer survivor succinctly recounted nature as ‘a place where I can get all together to myself’ [44].

Nature functioned as a platform for *social connections* bringing patients, friends and family together and helped peer bonding amongst cancer survivors who participated in structured nature activities. Gardens in care settings represented ‘somewhere to sit and laugh without disturbing others’ and a place for ‘playing, eating and being together’ [28]. One study found that the hospital garden promoted a ‘sense of belonging, support, and community’, where patients could ‘give and gain support’, ‘thrive in relaxed and unscripted conversation’ and engage in ‘social networking’ [45]. Ray and colleagues described how breast cancer survivors gained social support when connecting during a season of dragon-boating: ‘such a challenge provided them strength, promoted togetherness and offered support in facing their own fear of recurrence’ [44]. Similar outcomes were found for adolescent cancer survivors who ventured outdoors together and reported: ‘we became a family, we did things together … we realized it is easier to work together than work alone’ [13].

*Connection with nature* itself was valued and could enrich daily routines. Some participants ‘gained new personal perspectives towards nature’ [54], and ‘paid more attention to nature’ [46] after cancer. In this context, Unruh and colleagues found that nature experiences related to some participants’ conceptualizations of a higher power. For example: ‘you become very close to God with the blue sky and the feel of the earth under your feet. And you connect with nature, and your body becomes part of nature’ [41].

Another descriptive theme emerged in six documents (Table 2) showing that nature could connect participants with their *personal pasts* by evoking childhood memories [28, 45] or reminding of other past experiences such as ‘memories of trips, and places’ and ‘significant people and events’ [41]. This was not always found to be positive. One participant recalled her ‘memory plants’ reminding her of difficult relationships, which prolonged ‘unhappy memories’ [46].

Importantly, the notion of *losing connection* emerged from eight accounts in three separate studies denoting the experience of loss due to cancer progression and limited mobility or lifespan. Cancer related changes and restrictions could impinge on valued nature activities as illustrated by the following experience: ‘I felt very comfortable in my garden …. It was almost like a bond here, and it’s not there any more’ [40]. Another study found that, for patients who value gardening, losing ‘the capacity to garden can be very painful’ [41].

#### Being elsewhere, seeing and feeling differently

It was evidently important for participants to gain relief from immediate burden and to find different ways of conceptualizing their cancer experiences. Nature was a welcome temporary escape and could *distance patients from the strain* and, at times, unnecessary discomfort imposed by clinical settings and procedures. Butterfield described the hospital garden as offering ‘respite from the exhaustion of diagnosis, appointments and treatment’ [28].

A subtle distinction was made between gaining crucial distance from daily burden and the need to evoke a different state of mind through ‘physical and emotional *contrast’* [28]. The hospital garden actively contrasted the hospital’s anaesthetic qualities and counterbalanced ‘the large scale, highly mechanised, institutional, built environment’ [28].

Rowlands and colleagues demonstrated patients’ use of nature for *visual escape* from hospitalization when restrictions did not permit direct, embodied contact with nature. In their study with palliative cancer patients, they uncovered the value of connection with the outside world and recommended views ‘from the ward as well as the provision of large windows to allow a view from the bed areas’ and the ‘use of artwork depicting scenes of nature’ [11].

#### Exploration, inner and outer excursions

Overlapping the previous two themes of *connecting* and *being elsewhere* was the notion of exploring scenarios related to patients’ shifting inner and outer lives. It was evident that participants used nature to, individually and together, explore the consequences of their cancer. These explorations expressed the need to not only recover a sense of normality but discover new states, activities and behaviours.

*Extraordinary* nature experiences and *distant* locations had the potential to renew vigour and shift patients’ outlook. Two studies investigating experiences of outdoor activities in remote settings [13, 54] showed how play with proportionality provided a context large enough in which to place the extraordinary event of cancer diagnosis and approach new perspectives. These participants reported feeling exhilarated, proud, personally valued, increased self-esteem and self-empowerment, and a sense of succeeding.

The concept of exploration differed from merely getting away and included nuances of searching new ground and contemplating *future scenarios*. For example, the outdoor adventure study reported how the program could become a future source of ‘wonderful memories’ for participants to draw on when facing ‘any life challenges’ [13]. Nature also invited contemplation about uncertain future scenarios and life’s ending. For example: ‘It’s very possible it’ll [cancer] come back again and it’s possible it won’t … You put one [plant] in, sometimes she does, sometimes she doesn’t’ [46]. One study reported how a patient with uncertain prognosis used her garden to prepare for a future without her: ‘her garden would nurture others if she was no longer there … to garden even for a future without her … for people she loved.’ [41].

*New ideas and behaviours* could be explored through nature. Participants in a harvesting program were inspired to look for new recipes and try different produce resulting in greater vegetable consumption [45]. Similarly, learning about gardening was a welcome challenge and fostered creativity by ‘viewing the garden imaginatively or by actively gardening’ [46].

Gardening patients claimed a new sense of responsibility when *becoming garden caregivers* and committed to continued care of the garden after their study participation [45]. One patient explained that her caring for plants became a ‘marker of how far she had come since her diagnosis’ [46].

#### Home and safe

The theme *home and safe* appeared across eight documents (Table 2) and captured nature’s role as a ‘holding space between the inner more private or personal and the outer more public domain’ [28]. In the clinical setting, nature espoused qualities of safety and protection and provided a ‘secure comforting place’, ‘a sense of protection, refuge or sanctuary’, and a place that was ‘safe and secure and away from all the horrible experiences on the wards’ [28].

Participants articulated that their lives as cancer patients involved feelings of uncertainty, overwhelm, anxiety, and isolation. Interaction with the clinical environment intensified negative states and signified threat to privacy, personal control and, not in the least, life itself. Gardens were associated with privacy, safety and, most notably, a scaling down of the clinical to the *domestic* [28]. One participant reported remastering a sense of control through gardening: ‘At a time where … you are losing control over your life, over your future plans, over your bodily function, [gardening’s] something that you can control a little bit’ [46]. Butterfield described this role as a ‘screen or shield’ that protected from the overall harshness of the clinical environment [28]. Participants reported numerous interlinking qualities related to garden spaces within hospitals such as calming, relaxing, reassuring, strengthening, warming, inviting, containing, peace-giving, and easing (see Additional file 2 Themes and illustrative quotes).

Gardens provided consolation in the clinical setting and conveyed a sense of *caring and being cared for*. Inspiration and hope were instilled by gardens that were expressively well-maintained and cared for, and which, in turn, conveyed ‘an environment where people are caring’ [28].

Importantly, participants explained that nature could only take on a supportive role if they felt safe and near to clinical support. Participants wanted to feel close to, but not abandoned to natural settings, which was possible when these opportunities were well integrated into the clinical *infrastructure*. Such safely accessible nature spaces were described as an ‘escape from the hospital ward without going far’ [28], and were cast in phrases such as ‘stepping-stones’, ‘interim spaces’, and ‘buffer zones’ [28].

#### Symbolism, understanding and communicating differently

In seven studies participants repeated statements about using nature symbolism to better understand and communicate how their life situations had been changed by cancer. ‘Experiencing the garden as a living system’ [41] allowed a *metaphorical approach* to reassembling old and integrating new life components. Nature offered rich metaphors to capture these creative and adaptive processes. For example: ‘participants drew symbolism from the gardens, which they related to their own state of being or more specifically to their experiences of cancer and the so-called ‘cancer journey” [28]. Solace was found in life’s analogous unfolding with nature’s cycles. Being ‘symbolic of life and renewal in the life cycle’ [46], nature inducted patients to the states of life they were confronting. For example: ‘The garden also provided participants with an opportunity to be involved with the life cycle …. For some participants the garden was central to the struggle for life against cancer’ [54]. Participants consistently used this metaphor to situate their own stories into resonant contexts. In Butterfield’s study one participant noted: ‘It is also nice to look at something living … when you are trying to focus on surviving’ [28].

In some instances, participants recognized *themselves reflected and mirrored* in nature. Outer objects could resemble shapes of patients’ shifting inner lives. New meaning was made when recognizing aspects of their lives embodied and externalized in the material world. For example, when observing the ‘gesture of the plant’, one participant explained its ‘lovely quality of sadness’ and she found ‘it terribly important to have reflection on what’s happening inside’ [28]. Butterfield summarized that the garden could reflect ‘the visitor’s own experience as a cancer patient’ and found that some patients ‘paralleled their own existence, vulnerability, and survival to that of the natural environment’ [28]. Self-mirroring was not always reported as a positive experience. Some patients found it difficult and even unacceptable when nature triggered thoughts about possible futures: ‘I now get depressed when winter approaches …things dying, and I connect to that, and I’ve got to really fight that one’ [54]. Unruh discovered similar tensions and reported one patient’s challenging nature experience when ‘seeing the fragility of her plants’ lives reminded her of the fragility of her own life’ [46].

#### Benefitting from old and new physical activities

A theme identified in six studies related to the benefits emerging from continuity with pre-cancer physical activities and from adopting new activities. *Maintaining enjoyable activities* could strengthen ties with normality and sustain positive health behaviour. For example, a seasoned gardener described ‘a life-long appreciation of nature’ and continued gardening after cancer [46]. Similar sentiments were expressed by a patient who identified gardening as an ‘intensely enjoyable and familiar’ part of life [41].

Adopting *new activities* was helpful when attempting to break away from cancer related experiences. Participants prevented from maintaining their home gardens due to post-treatment fatigue welcomed the opportunity to try community gardening instead [45]. Likewise, novel outdoor adventures provided a myriad of positive experiences [13].

Nature activities including gardening and dragon-boating resulted in physical *benefits* such as adopting healthier diets [43, 45], increased physical activity [28, 45], improved fitness, and ‘loosened joints’ [44]. Overall, benefits extended beyond the physical dimension and, in participants’ own words, included: ‘buffer against stress’ [44], ‘the courage to exist and be human’ [13], ‘sense of satisfaction and accomplishment’, ‘energized and renewed’, ‘source of relaxation’ [46], and ‘spiritual and emotional strength’ [40].

#### Enriching aesthetic experiences

It was apparent that for a significant number of participants nature provided enrichment through *stimulating* and enlivening their physical senses.

Nature’s rich materials offered ‘contrasts of colour, texture, scale, fragrance and season’ and was reported to ‘engage the senses in a different way’ offering ‘soothing, calming, but also lively, contrast’ to the clinical environment [28]. In particular, water was mentioned for its therapeutic, soothing and calming qualities. Nature tapped the immediacy of the senses and was something tangible to orient towards, an ‘external stimulus … for restoring a sense of peace and aliveness’ [44]. One participant recollected nature’s quickening qualities during a cycle of chemotherapy: ‘air was so fresh, everything was so fresh, it was alive’ [46]. Those who identified as gardeners found their practice enriched when creatively playing with landscape features and enjoying their ‘visual and tactile pleasures’ [46]. Interestingly, nature could also provide ‘sensory quietness’ [28] such as softness, gentleness, and shielding from unwanted stimuli. The chance to ‘hear silence … wordlessness’ [28] was amongst the sensory relief sought in hospital gardens.

Patients who felt *aesthetically enriched* by nature often reported experiences of appreciating its beauty, peace, tranquillity, and the solitude found in nature (see Additional file 2 Themes and illustrative quotes).

### Overview of findings

The present synthesis found that cancer patients valued contact with nature and benefitted from opportunities to connect with nature. Engaging with nature eased the strain related to cancer diagnosis and treatment by taking on several supportive roles: facilitating valued connections, transporting away from the burden and threat of cancer, encouraging inner and outer explorations, offering safe refuge, providing metaphoric material for understanding life changes, motivating physical activity, and enriching cancer patients’ lives aesthetically.

## Discussion

The aim in this review was to describe and meaningfully synthesise the range of nature experiences reported from the cancer patient perspective and to discern its relevance in cancer patients’ lives. While exploring vastly different levels of nature engagement, the included studies’ common topic revealed overlapping layers (themes) of the shared human phenomena that occur when a person affected by cancer engages with nature. The findings shed light on the initial questions the study set out to address: how nature supports or detracts from cancer patients’ recovery experiences (does it work?); and if patients value these opportunities (should it be used?). The seven identified themes explored the values held by patients who used nature to address some of their needs. These needs included: maintaining continuity with surroundings and activities, a sense of normality and control over one’s life, social support and integration, community participation, occupational and leisure engagement, access to a familiar support structure, creating meaning and perspective, physical activity, and aesthetic and sensory enrichment. Nature represented an unburdened and uninterrupted space embedded in everyday life from which patients sourced strength and meaning to address these needs. The benefits shown here extend across bio-psychosocial dimensions, which correspond with supportive care needs identified in previous research [17, 18]. These known impacts of cancer reveal patients’ sudden struggle and vulnerability when tasked to navigate the imminent and ambient challenges of daily living.

The present findings contribute to discourse in psycho-oncology investigating patients’ need to respond to cancer’s urgent threat by constructing new ways of handling life and accepting a “new-normal” [56]. Drawing on Attachment theory [57], it is theorized that secure attachment to a supportive structure or “helping system” [58] can support patients’ stepwise process of accepting lives shaped by cancer. It is premised that when attached to a “secure base” [57], patients are enabled to risk exploring various real and imagined future scenarios and approach a shifting normality that now includes their cancer experiences. Salander [59] suggests the application of the Winnicottian “intermediate area” [60] for interpreting cancer patients’ mental coping manoeuvres in this process. From this perspective, the construction of a private place between inner and outer reality unburdens patients from practical demands and immediate here-and-now reality, allowing a more creative approach to dealing with their situations. The present meta-synthesis found that nature could be seen as a potential “secure base” offering patients a familiar and nurturing context from which new perspectives can emerge and caring connections can be made with themselves, others, the past, and the future. The findings show that nature provided patients with unburdened physical and psychic space that was regarded a valued component of everyday life and invested with personal significance. As such, nature supported patients’ inner and outer manoeuvres to navigate the clinical and personal consequences of cancer.

### Practical implications

Comprehensive cancer care services need to consider patients’ values and experiences. Qualitative research is designed to generate a deep and broad understanding of human experiences and processes. Meta-synthesis can improve the translation of qualitative research into practice through locating, condensing and appraising relevant findings for the medical readership and healthcare management who govern clinical practice, research, and policy [61].

Patients’ motivations to seek nature, and the effects of these interactions were highly personal. The idiosyncratic associations between type of engagement and outcomes suggest that the benefits derived from engaging with nature cannot be predetermined nor administered. Contrary to what an interventionist approach would suggest [62], there is no indication to utilize nature in a prescriptive manner. However, cancer patients consistently attributed importance to engaging with nature and derived, in various forms, benefit and meaning from these interactions. The findings give credence to validating and enabling cancer patients’ own resources by appraising aspects of their lives and histories from which they draw meaning, strength, and hope. A range of practical examples from the collected literature suggest pathways for patients to access nature experiences, both in cancer care and home environments. Views to nature and nature art are easily incorporated in the design of clinical settings if put on the agenda, which patients reportedly derive benefit from. Hospital courtyards and home gardens can encourage various degrees of physical activity, motivate fruit and vegetable consumption, and create opportunities for connecting socially. Organized outdoor trips and activities can powerfully connect patients on a peer level and motivate positive lifestyle behaviours. While many examples are unsurprisingly simple and commonplace, further research is required to identify, understand, and safely implement such additional care opportunities for their maximum benefit.

### Limitations

The findings relate to cancer patients in westernized countries and cannot be said to transfer to other clinical or geographic populations. Although this review aimed at comprehensive sampling, the dearth of relevant literature resulted in a very small sample size (*n* = 11 documents), with a correspondingly small combined participant sample (*n* = 240). Based on Sandelowski’s recommendation that large sample size (exceeding 10 studies) can compromise “deep analysis” and “threaten interpretive validity” in meta-synthesis [29], the found literature was deemed sufficient to proceed with the synthesis.

Many of the included studies have serious methodological limitations, which must be considered when assessing the credibility of this review’s findings. In particular, the absence of audit trails, negative case reporting and researcher reflexivity challenge confirmability and present the possibility of bias in the recruitment and research procedures. To increase the review’s credibility, focus was placed on raw data (participants’ words) in order to keep the interpretations closer to participants’ own experiences.

Lastly, this review lacked an inter-rater process (such as member checking or peer debriefing), which is recommended in qualitative research to aid interpretation bias in work undertaken by sole researchers [42, 63]. While single author quantitative meta-analyses and qualitative meta-syntheses are uncommon, neither are unprecedented, see for example Dijkers [64] and Hammell [23]. Strategies were employed to mitigate this shortcoming and increase credibility. Firstly, it is clearly stated in the Methods section that this study represents a sole researcher study preparing the reader to navigate potential sole author bias. The findings remain traceable to their source materials by providing a considerable amount of primary data (audit trail) for internal validation and to enable the reader’s own audit of interpretations. Furthermore, detailed documentation of the search procedures and quality appraisals is made available to reflect rigour and the inclusion of topically relevant studies.

## Conclusions

Awareness of cancer patients’ nature experiences can enrich communication between clinician and patient, broaden recommendations for health behavior, and guide the design of care settings and services. Through hearing the patient’s own voice, supportive cancer care can align with meaningful and relevant aspects of patients’ lives and offer effective care. This meta-synthesis contributes to the larger process of hearing and communicating patient values across sectors. Sensitizing research and practice fields to these issues may inspire different approaches to asking questions, listening carefully, and delivering care.

## Additional files

Additional file 1: Search protocol. (PDF 46 kb)

Additional file 2: Themes and illustrative quotes. (PDF 85 kb)

Additional file 3: Excluded publications. (PDF 77 kb)

Additional file 4: Quality assessment. (PDF 35 kb)

## Abbreviations

ACTAHORT: Acta Horticulturae International Society for Horticultural Science
AHTA: American Horticultural Therapy Association
CASP: Critical Appraisal Skills Programme
CINAHL: Cumulative Index to Nursing and Allied Health Literature
COREQ: Consolidated Criteria for Reporting Qualitative Research
JBI-QARI: Joanna Briggs Institute Qualitative Assessment and Review Instrument
UK: United Kingdom
US: United States
