# Supplementary material for: The role of nature in cancer patients' lives: a systematic review and qualitative meta-synthesis
Source: BMC Cancer. 2017 May 25;17:370. doi: 10.1186/s12885-017-3366-6 (PMC5445345; doi:10.1186/s12885-017-3366-6)
Supplement: Supplementary file 1 — Search protocol. (PDF 46 kb) [file 12885_2017_3366_MOESM1_ESM.pdf]

Additional file 1 Search protocol

EBSCOhost: CINAHL & PsycInfo

Cluster 1: Exp Neoplasms/
OR
cancer* or neoplasm* or tumo* or carcinoma* or malignanc* or leukemia* or leukaemia* or oncolog* or metastas*

AND

Cluster 2: garden* or horticultur* or wilderness or forest* or woodland*
AND
"natur* based" or "natur* assisted" or "urban green*" or "green care" or "benefit* of natur*" or "natur* setting*" or "natur* environment*" or "outdoor nature" or "interacting with nature" or "nature experience*" or "experiencing natur*" or "nearby nature" or "environmental intervention" or "physical environment feature" or environment* impact" or healing environment*" or "outdoor* environment*" or "indoor plant*" or "window view*" or "restore attention" or "attention restoration" or "distraction therapy" or "distraction techniques" or " outdoor adventure" or "therapeutic camping" or windowless or "green park*" or "urban park" or "nature based sound"

Ovid: MEDLINE

Cluster 1: Exp Neoplasms/
OR
cancer* or neoplasm* or tumo* or carcinoma* or malignanc* or leukemia* or leukaemia* or oncolog* or metastas*

AND

Cluster 2: garden* or horticultur* or wilderness or forest* or woodland*
OR
"natur* based" or "natur* assisted" or "urban green*" or "green care" or "benefit* of natur*" or "natur* setting*" or "natur* environment*" or "outdoor nature" or "interacting with nature" or "nature experience*" or "experiencing natur*" or "nearby nature" or "environmental intervention" or "physical environment feature" or environment* impact" or healing environment*" or "outdoor* environment*" or "indoor plant*" or "window view*" or "restore attention" or "attention restoration" or "distraction therapy" or "distraction techniques" or " outdoor adventure" or "therapeutic camping" or windowless or "green park*" or "urban park" or "nature based sound"

Search Limits: English language and humans and yr="1985 -Current

Cochrane Database of Systematic Reviews

neoplasms
OR
cancer* or neoplasm* or tumo* or carcinoma* or malignanc* or leukemia* or leukaemia* or oncolog* or metastas*

AND

garden* or horticultur* or wilderness or forest* or woodland* or natur* or environment* or park* or "urban green*" or "green care" or "indoor plant*" or window* or "restore attention" or "attention restoration" or "distraction therapy" or "distraction techniques" or outdoor* or "therapeutic camping"

Search Limits: Publication Year from 1985 to 2015 (Word variations have been searched)
