# Supplementary material for: The role of nature in cancer patients' lives: a systematic review and qualitative meta-synthesis
Source: BMC Cancer. 2017 May 25;17:370. doi: 10.1186/s12885-017-3366-6 (PMC5445345; doi:10.1186/s12885-017-3366-6)
Supplement: Supplementary file 2 — Themes and illustrative quotes. (PDF 85 kb) [file 12885_2017_3366_MOESM2_ESM.pdf]

Additional file 2 Themes and illustrative quotes

	Core and descriptive themes	Illustrative quotes: Examples of raw data from primary sources (author / year)
1	**Connecting with what is valued**<br>Connecting with something outside	"I find nature very healing. So, if people need to heal, they need to get out of the city, and get into nature. Nature being water, animals, trees. … But it just makes me feel good. Makes me feel good to be there. (Nancy)" (English 2008); "… it helps you appreciate and take hold of everything every day and you don't take it for granted anymore. (P11)" (Ray 2013); "… taught me how to grow my own fruits and vegetables and the importance of eating more fruits and vegetables, but also to focus on the circle of life. … a more optimistic outlook on life in general…" (Blair 2013)
	Connecting with Self	"... I just find it is a place for me to let go of everything and when on the water I am removed in some way from the land. I just find it is a place where I can get all together to myself. (P04)" (Ray 2013);
	Connecting with others	"… calming, it is beautiful, and it is just really wonderful. Being together is uplifting, it is a sunny, beautiful day, it is warm, it is glorious, you have worked, you are tired, you get out from the water, you are together, you are laughing, you are joking, you are putting out some physical effort and it lifts you up..." (Ray 2013); "… social networking aspect of the garden, from meeting new survivors to conversing with acquaintances and students. … the garden as a meeting place where they could share ideas, give and gain support, and thrive in relaxed and unscripted conversation." (Spees 2015); "Ensemble … We became a family, we did things together, we played together … (Participant 3, tape 25, p 5)." (Stevens 2004)
	Connecting with nature	"… gained a new personal perspective towards nature after experiencing a breast cancer diagnosis. Four women discussed feeling closer to nature as a result of their experience with breast cancer" (English 2008); "Paying more attention to nature through interaction with plants and animals was sometimes heightened because of the experience of having cancer." (Unruh 2000); "… feeling connected to their garden in some way. Gardeners spoke about feeling 'close to nature' or having a bond between themselves, their garden, and the birds and animals that visited the garden." (Unruh 2011)
	Connecting with the past, reminiscing and remembering	"… feelings of connectedness with the planet (i.e. earth and soil) and vivid memory recalls of early childhood experiences with gardening and farming." (Spees 2015); "… "memory plants" because they were gifts from friends or they had belonged to a special person. However, plants that prolong unhappy memories may languish." (Unruh 2000); "Participants at all case study sites spoke of how certain details or features of the gardens reminded them of other times and places. Their memories would be triggered by something as small as a flower bud or the texture of a leaf. Memories ranged from childhood experiences, holidays abroad or life at home." (Butterfield 2014)
	Losing connection	"The loss affected him on a deeply emotional, psychological and spiritual level because the garden had been so much a part of his inner being and self-identity. He said: Last year I felt very comfortable in my garden. … I don't feel that anymore. It was almost like a bond there, and it's not there any more. ..." (Unruh 2011); "During the last four to five months of her life, Karen took up a new leisure activity – day trips in a mobile home. Karen continued to enjoy looking at her garden, but she seemed increasingly detached from it as her health deteriorated." (Unruh 2002)
2	**Being elsewhere, seeing and feeling differently**<br>Gaining distance (break) from everyday strain	"… on days when you've had treatment, you may not feel so great ... [but] you can go out there ... and listen to the birds and look at the garden and maybe go into another world a little bit." (Unruh 2000); "… offered respite from the exhaustion of diagnosis, appointments and treatment." (Butterfield 2014); "… therapeutic qualities of nature located outside the realm of day-to-day life can positively contribute to psychological well-being. The calming and inspiring characteristics of different aspects of nature seem to contribute to emotionally uplifting experiences." (English 2008); "For stress levels I think it is great because you get out on the water and you forget. I don't think of my job, I don't think of cancer, I don't think of any problems." (Ray 2013)<br>"… improved their mental and emotional health by providing a reprieve from the everyday stresses of living as a cancer survivor."

		(Spees 2015); "… garden was frequently perceived as a refuge, an escape from the fear and worry associated with cancer, a place that was safe." (Unruh 2002)
	Contrasting the clinical experience	"… closeness appears to be part of their strength … a very strong physical and emotional contrast to the experience of the hospital. Visitors immediately move from the large scale, highly mechanised, institutional, built environment to one that is small-scale, domestic and personal." (Butterfield 2014); "When people are having treatment for cancer they feel 'people invaded' people are always doing something to you…Gardens offer free form." (Butterfield 2014); "… gardens offered was a chance to hear silence; not in the sense of noiselessness but rather wordlessness. … able to shut out all extraneous noise." (Butterfield 2014);<br>"… time in hospital can be an exhausting mix of endless waiting combined with the sense of having no time to oneself. that the gardens offered them different and often more soothing experiences of time." (Butterfield 2014)
	Visual escape, a different way of being elsewhere	"… look out in my backyard, that you know, it's a big backyard, it's very treed, it's very calming, and just that vista is something that I remember quite vividly … (Cindy)" (English 2008); "… sit, relax in a bit more comfort (In the day room). Look outside rather than at your wall all the time." (Rowlands 2008); "… too unwell to access outside found a view outside and natural pictures beneficial. … … provision of large windows to allow a view from the bed areas." (Rowlands 2008); "… spiritual and emotional strength to withstand the cancer, which had spread, from her garden. In the fall, when the garden became less accessible to her, she renovated her home to keep the garden visually present year round." (Unruh 2002); "… the presence and views of plants within the buildings helped people to feel calmer." (Butterfield 2014)
**3**	**Exploration, making inner and outer excursions**<br>Exploring the distant and extraordinary	"… extraordinary encounters with nature are also important for healing. Eight women spoke about the importance of nature in distant locations (e.g., cottages, vacation spots) …. Some travel to favourite destinations as a strategy to stay psychologically healthy and to take their minds off of the possibility of cancer recurrence." (English 2008); "Hiking and accomplishing their objective of climbing the mountain … Knowing they were able to participate in something so ''special'' made them feel valued as individuals …helped to rebuild their self-esteem." (Stevens 2004)
	Exploring future scenarios	"… might not be living in the next year. Her daughter helped her to understand that her garden would nurture others if she was no longer there. This thought helped Monica to garden even for a future without her; the garden would be for people she loved." (Unruh 2011); "Most poignantly, the beauty, tranquillity, and breathtaking sites they have experienced in the remote area are images they believe they will recall for years to come. The memory of being able to enjoy themselves, in spite of having cancer, is also significant …." (Stevens 2004); "… gardening was at times deeply connected to coming to terms with her own mortality: I just happened to notice lately, emotionally, how I was feeling as I looked at things turning yellow with the frost. … 'Oh, yes, I'm thinking about dying'. … as I watch this garden die, the two things kind of click together – my body and the garden. Because in reality, the garden is a metaphor for life …." (Unruh 2011)
	Exploring new ideas, behaviors and activities	"… they were exposed to several new vegetables (eg, purple kohlrabi, rainbow Swiss chard) and enjoyed experimenting with these new foods and recipes." (Spees 2015); "… cancer brought her in touch with nature. She said: Cancer does force you to stop and pause ... Now I've actually got time to contemplate nature ... you see the world of nature differently and that's because of cancer." (Unruh 2000); "… a creative outlet that allowed her the role of an artist who plants with a palette. … Brenda enjoyed the challenge of learning what would grow and prosper in her garden. … All three participants described lessons of life that were taught to them through their gardens." (Unruh 2000)
	Exploring the caregiver's role, caring for the garden	"…was this little teeny bird and it was really yellow and I had never seen it before and it had landed on my cosmos and my poor cosmos was leaned right over. … I watered it and watched it grow and there was this little bird on this delicate flower." (Unruh 2000); ""I may not always feel up to it, but … you better believe that I am not going to sit here and let them rot." (from a cancer

		survivor with multiple comorbidities)" (Blair 2013); "… a strong sense of responsibility and shared commitment to the garden and other survivors to continue harvesting throughout the season." (Spees 2015); "… gardening is active, interactive, and interconnected. The gardener contributes to the welfare of the garden, and the garden responds in the way it grows and develops." (Unruh 2011)
**4**	**Home and safe**<br>Domestic scale	"Participants said that the gardens contributed to the sense of containment and privacy. Not in the sense of blocking out or hiding from the outside world … a sense of protection, refuge or sanctuary. … gardens helped to screen or shield the harshness of the overall hospital experience." (Butterfield 2014); "Being enclosed makes you feel at peace and safe, it makes you feel calm and relaxed … you are free like a bird (SRPVF 48, 2010)" (Butterfield 2014)
	Caring and being cared for	"… specifically within a healthcare context a garden provides opportunities to emphasise and fine-tune the quality of caretaking within the place. … offers caretaking at a deeper level." (Butterfield 2014); "… a means by which other people were able to express their love and concern for the participants through giving plants, and by helping with the work of the garden." (Unruh 2002); "… gardens contribute to feelings of comfort in the sense of making people feel at ease. … also the gardens contributed to the sense that the place was about comfort and help." (Butterfield 2014)
	Supportive infrastructure	'‘… nature's so important to me … these trees can survive in all of this … the trees are good for us. … something that we need too for our health. (Rachel)" (English 2008); "… a sense of controllability in life during the illness experience.... you are losing control over your life, over your future plans, over your bodily functions, [gardening's] something that you can control a little bit." (Unruh 2000); "… gardens made the centres seem warm and inviting. … a good first impression and created a calming atmosphere …" (Butterfield 2014)
**5**	**Symbolism, understanding and communicating differently**<br>Using metaphors found in nature	"… through connecting with nature and viewing themselves in unity with the natural environment, participants also understood the natural environment as a metaphor for emotional insight." (Pascal 2010); "They were symbolic of life and renewal in the life cycle." (Unruh 2000); "Specific plants also often become symbols that have particular personal meaning for someone experiencing cancer." (Unruh 2000); "… feeling connected to a life force and witnessing survival and rebirth in the garden were poignant because as Florence stated, 'There is a lot of death in a garden'." (Unruh 2011); "… they are with us when we are in the ground, when we are out there doing something in the ground. Because that is what we are. We are living beings as they are. And it all sort of meshes together. But we forget that we are just a part of that living thing." (Unruh 2011); "… winter in the garden was as enjoyable as summer. This was because the "flow" of seasonal change emphasised the passing of time, the transience of all life and reaffirmed a key essence of living – constant change and growth. … the symbolism of certain plants to contemplating the cycle of life and death" (Butterfield 2014); "… understanding cancer; wondering about root causes, health, growth and mutations. Here participants drew on the symbolism of plants and their historical and cultural associations." (Butterfield 2014)
	Reflecting and mirroring inner and outer life	"… they related to their own state of being or more specifically to their experiences of cancer and the so-called 'cancer journey'." (Butterfield 2014); "This has sadness that I love. It's the gesture of the plant. It's very beautiful - it have a lovely quality a sadness. … It terribly important to have reflections on what's happening inside – to have something that is already connecting to our feelings."(Butterfield 2014); "I now get depressed when winter approaches … slow-down and things dying, and I connect to that, and I've got to really fight that one. I almost need to get away and see sun." (English 2008); "I'm closer to nature than I was before … you're alive, I'm alive, you know, that kind of closeness, that proximity. (Victoria)" (English 2008);<br>"… life mirrored in plants given to her during her illness. She kept these plants as a marker of how far she had come since her diagnosis … (Unruh 2000); "... there's life and death, there's beauty and there's patience, being patient because maybe the flowers you put in aren't going to thrive ... You may think you are in control of it (the garden), but you have the control to set the garden …" (Unruh 2000); "… an opportunity to be involved with the life cycle in a way that was intimate and within their control."

		(Unruh 2002); "I think it shows me the fact that there is hope. … It does happen in nature .... (Unruh 2011); "It's sort of an extension of me, in putting my mark if you will, here. It's sort of expressing some of my joy. (Lilly)" (Unruh 2011)
6	**Benefitting from old and new physical activities**<br>Enjoying new and old activities in nature	"All of the participants plan to continue gardening and most even planned to expand their garden." (Blair 2013); "Physically, participants spoke of appreciation for the many fitness and health benefits of active participation during the season, and also of the importance of maintaining fitness year-round." (Ray 2013); "I find being on the water is that added benefit and it is very peaceful to be out there and I so look forward to it. It is addicting! ... " (Ray 2013); "… harvesting at the garden encouraged them to engage in more physical activity than they would have otherwise. Many expressed that because they felt more energized after consuming greater amounts of garden produce, they had further incentives to keep engaging in the physical activity required to harvest more produce." (Spees 2015); "The garden and gardening was simply an intensely enjoyable and familiar part of their lives. Much of their day was structured about the garden." (Unruh 2002)
	Benefitting from being active in nature	"… gardening experience motivated them to eat a healthier diet overall and to eat more vegetables in particular." (Blair 2013); "… improved cardiovascular endurance, more strength in arms, shoulders, legs and core, and the positive impact of improved fitness levels on daily functioning. (in reference to dragon boating)" (Ray 2013); "… the bounty of fresh produce enhanced their ability to consume a primarily plant-based diet while reducing overall intakes of red meat and highly processed foods." (Spees 2015); "Survivors reported improvements in both mental and physical health that increased over the course of the harvesting season." (Spees 2015); "… helped women stay in the 'here and now' instead of ruminating about the past or focusing on stressful events. … finding unison of stroke with team-mates, and enjoying 'being in the present' was described as a powerful means of managing stress." (Ray 2013); "Just getting out there and picking, and even helping others pick, getting your hands dirty, really, mentally helps some of that stress …" (Spees 2015); "So at this level (in reference to outdoor expedition), yes, it gives them the courage to have the courage to fight, the courage to exist and be human." (Stevens 2004); "… the sense of satisfaction and accomplishment they experienced through their gardening efforts." (Unruh 2000); "… got rid of a good deal of angst, anger, and frustration by going out and weeding."" (Unruh 2000)
	**Enriching aesthetic experiences**<br>Stimulating sensory experiences	"… valued the contrasts of colour, texture, scale, fragrance and season within the gardens. They spoke about their enjoyment of the 'smooth and prickly – the sensory stuff'" (Butterfield 2014); "… importance of fragrant plants for many of the gardens' users. … there were no negative comments about smell. Participants talked about the pleasure of sniffing a particular bud, brushing past a herb or simply enjoying the smell of outdoors." (Butterfield 2014); "I love the fact that the plants are scented. The garden tickles all your senses." (Butterfield 2014); "… bold colourful planting, which they saw as dynamic and joyful." (Butterfield 2014); "… sounds specific to the gardens, such as birdsong, insects, rustling grasses or trickling water." (Butterfield 2014); "… joy of being in nature, the beauty, the smells, the sounds and the feeling of the paddles moving in unison going through the water." (Ray 2013); "… following her chemotherapy, said, "I loved being outside because the air was so fresh, everything was so fresh, it was alive." (Unruh 2000); "… environment is so calming, so therapeutic for me. Sea air, I just, I crave the air, because the smell of it has a great effect. (Victoria)" (English 2008)
	Aesthetic enrichment	"… it's lovely to be able to sit here and look out, enjoy the bird life, the kangaroos, the bugs, the swallows." (Pascal 2010); "The interaction with nature was expressed as being an external stimulus that was important for restoring a sense of peace and aliveness." (Ray 2013); "Notably, the beauty of the scenery was continuously described as "amazing" and "breathtaking."" (Stevens 2004); "The solitude, tranquillity, and beauty of the surroundings allowed them time to reflect and become more in touch with themselves." (Stevens 2004)
