# Supplementary material for: The role of nature in cancer patients' lives: a systematic review and qualitative meta-synthesis
Source: BMC Cancer. 2017 May 25;17:370. doi: 10.1186/s12885-017-3366-6 (PMC5445345; doi:10.1186/s12885-017-3366-6)
Supplement: Supplementary file 3 — Excluded publications. (PDF 77 kb) [file 12885_2017_3366_MOESM3_ESM.pdf]

Additional file 3 Excluded publications

Author (Year)	Topic	Reason for exclusion	Source
Abrams (2006)	Role of gardening when dealing with cancer	Not peer-reviewed	CINAHL + PsycInfo
Baird and Bell (1995)	Relevance of a window view during hospitalization (leukemia patient)	Theoretical exposition	Snowballing
Balen (1998)	Issues of providing an outdoor activity week (pediatric cancer patients)	Quantitative research with minor and unclear qualitative component	Snowballing
Bekesi (2011)	Impact of camping experience on HQOL (chronic disease patients)	Mixed clinical populations	Snowballing
Benson (1987)	Impact of camping experience on Self-Concept (pediatric cancer patients)	No data concerning nature	Snowballing
Block (2004)	Role of optimal healing environments in oncology	Theoretical exposition	OVIDMedline
Bluebond-Langer (1990)	Impact of camping experience on perceptions of cancer and treatment (pediatric cancer patients)	No data concerning nature	Snowballing
Cimprich (2003)	Environmental intervention for attention restoration (newly diagnosed breast cancer)	Not qualitative research	OVIDMedline
Conrad (2009)	Impact of camping experience on social support and adjustment (pediatric cancer patients)	No data concerning nature	Snowballing
Dahlquist (2002)	Distraction during surgical procedures (pediatric cancer patients)	Electronic toys, not nature related	OVIDMedline
Epstein (2004)	Adventure therapy for mental health promotion (pediatric cancer patients)	Description of intervention only	OVIDMedline
Epstein (2004)	Adventure therapy for adolescents cancer patients	Description of intervention only	Snowballing
Fillon (2014)	Gardening as therapeutic intervention for cancer patients	Not peer-reviewed	OVIDMedline
Fried (2008)	Therapeutic horticulture program for cancer patients	Lacks methodological rigour and primary data	CINAHL + PsycInfo
Greenleaf (2014)	Nature-based counseling in practice	Not cancer related	Snowballing
Hancock (2011)	Impact of camping experience for siblings (pediatric cancer patients)	Focus on family of patients not cancer patients	Snowballing
Jankovich (1994)	Communicating diagnosis with garden analogy (leukemia pediatric patients)	Lacks methodological rigour and primary data	Medline
Jessee (1986)	Nature experiences for hospitalized children	Not cancer related	Snowballing
Kessell (1985)	Adventure therapy for chronically ill and disabled youth	Not cancer related	Snowballing
Kiernan (2002)	Children’s perspectives of therapeutic recreation	Mixed clinical populations, not clearly separated	Snowballing
Kiernan (2005)	Children’s perspectives of therapeutic recreation	Mixed clinical populations, not clearly separated	Snowballing
Kinsella (2006)	Safety of summer camp for children with chronic and/or life threatening illness	Mixed clinical populations	Snowballing
Lane (2005)	Physical effects of dragon-boat training (breast cancer)	Physical activity research	Snowballing
Lange (2010)	Outdoor adventures for young adults with cancer	Not peer-reviewed	Snowballing

Li (2009)	Effect of phytoncide from trees on human natural killer cell function	Participants were not cancer patients	OVIDMedline
Li (2010)	Physiological effects of day trip to a forest park (NK activity, expression of anti-cancer proteins)	Participants were not cancer patients	OVIDMedline
Li (2011)	Effect of forest environments on human natural killer (NK) activity	Participants were not cancer patients	OVIDMedline
Lombard (2014)	Perceptions of gardening to prevent diabetes and cancer	Not relevant	OVIDMedline
Martiniuk (2004)	Camping programs for children with cancer and their families	Literature Review, used for snowballing	Snowballing
McDonough (2008)	Psychosocial effects of dragon boating (breast cancer survivors)	Focus on body image and social support	Snowballing
McKenzie(1998)	Effects of dragon boating (breast cancer)	Physical activity research	Snowballing
Meltzer (2004)	Camping for chronically ill children, respite care for mothers	Mixed clinical populations	Snowballing
Mitchell (2002)	Effects of dragon boating (breast cancer)	Physical activity research	Snowballing
Mitchell (2007)	Effects of dragon boating (breast cancer survivors)	Physical activity research	Snowballing
Morrison (2011)	Pandemic (H1N1) outbreak during oncology camping	Not relevant	Snowballing
Nakau (2013)	Intervention in urban green space for cancer patients	Not qualitative research	OVIDMedline
Packman (2004)	Psychological effects of camping for siblings (pediatric cancer patients)	Focus on family of patients only	Snowballing
Park (2002)	Effects of plants on pain tolerance in a simulated hospital room	Not cancer related	ACTAHORT
Park (2009)	Impact of plants in hospital rooms on recovering from surgery	Not cancer related	ACTAHORT
Parry (2008)	Effects of dragon boating (breast cancer survivors)	Physical activity research	Snowballing
Rogers (2011)	Physical activity impact on fatigue and depression (breast cancer survivors)	Not qualitative research	OVIDMedline
Rosenberg (2014)	Impact of adventure program on body image and psychosocial functioning (young adult cancer patients)	Not qualitative research	OVIDMedline
Saadatmand (2012)	Effect of nature-based sounds in hospital patients	Not cancer related	Snowballing
Sabiston (2007)	Impact of dragon Boating on positive psychological growth (breast cancer survivors)	Physical activity research	Snowballing
Sahler (1989)	Impact of camping program for siblings (pediatric cancer patients)	Focus on family of patients not cancer patients	Snowballing
Sahlin (2012)	Experiences of nature-based therapy in rehabilitation	Participants were not cancer patients	AHTA
Smith (1987)	Impact of camping experience on family life (pediatric cancer patients)	Focus on social interaction and physical activity	Snowballing
Taft (2004)	Therapeutic horticulture program for cancer patients	Not qualitative research	AHTA
Taft (2007)	Therapeutic horticulture program for cancer patients	Lacks methodological rigour and data from health professionals not separated from cancer patients	AHTA
Tӧrӧk (2006)	Effectiveness of therapeutic camping (adolescent cancer patients and diabetes patients)	No data concerning nature	Snowballing

Wellisch (2006)	Psychosocial impacts of camping experience (pediatric cancer patients and siblings)	Not qualitative research	Snowballing
Whitehouse (2001)	Evaluating oncology garden environment	Post occupancy evaluation.	Snowballing
Wu (2011)	Experiences of oncology camping (pediatric cancer patients and siblings)	No data concerning nature	Snowballing
Wynn (2012)	Experiences of adventure therapy (adolescent cancer survivors)	Lacks methodological rigour and primary data	CINAHL + PsycInfo
Yamane (2002)	Physiological and emotional effects of indoor horticultural activities	Not cancer related	ACTAHORT
Young-Mason (2008)	Garden interventions for cancer patients	Lacks methodological rigour and primary data	OVIDMedline
Young-Mason (2010)	Role of plants and art in cancer care	Theoretical exposition	CINAHL + PsycInfo
