# Supplementary material for: The role of nature in cancer patients' lives: a systematic review and qualitative meta-synthesis
Source: BMC Cancer. 2017 May 25;17:370. doi: 10.1186/s12885-017-3366-6 (PMC5445345; doi:10.1186/s12885-017-3366-6)
Supplement: Supplementary file 4 — Quality assessment. (PDF 35 kb) [file 12885_2017_3366_MOESM4_ESM.pdf]

Additional file 4 Quality assessment following COREQ guidelines

	COREQ (Tong et al 2007)			
**First author (year)**	**Domain 1 (8 items) Research team and reflexivity**	**Domain 2 (15 items) Study design**	**Domain 3 (9 items) Analysis and findings**	**Total (32)**
Blair (2013)	0	6	1	7
Butterfield (2014)	8	9	5	22
English (2008)	0	9	6	15
Pascal (2010)	0	9	6	15
Ray (2013)	0	10	7	17
Rowlands (2008)	0	6	5	11
Spees (2015)	1	10	8	19
Stevens (2004)	7	12	7	26
Unruh (2000)	0	10	7	17
Unruh (2002)	4	9	6	19
Unruh (2011)	5	10	8	23
